# A Systematic Review of the Clinical Value and Applications of Three-Dimensional Printing in Renal Surgery

**DOI:** 10.3390/jcm8070990

**Published:** 2019-07-08

**Authors:** Catalina Lupulescu, Zhonghua Sun

**Affiliations:** Discipline of Medical Radiation Sciences, School of Molecular and Life Sciences, Curtin University, Perth 6102, Australia

**Keywords:** renal disease, renal cell carcinoma, renal tumour, three-dimensional printing, surgical planning, model, simulation

## Abstract

The purpose of this systematic review is to collate and analyse the current literature which examines clinical applications of 3D printing for renal disease, alongside cost and time duration factors associated with the printing process. A comprehensive search of the literature was performed across five different databases to identify studies that qualitatively and quantitatively assessed the value of 3D-printed kidney models for renal disease. Twenty-seven studies met the selection criteria for inclusion in the review. Twenty-five were original studies, and two were case reports. Of the 22 studies reporting a qualitative evaluation, the analysis of findings demonstrated the value of the 3D-printed models in areas of clinician and patient education, and pre-surgical simulation for complex cases of renal disease. Of five studies performing a quantitative analysis, the analysis of results displayed a high level of spatial and anatomical accuracy amongst models, with benefits including reducing estimated blood loss and risk of intra-operative complications. Fourteen studies evaluated manufacturing costs and time duration, with costs ranging from USD 1 to 1000 per model, and time duration ranging from 15 min to 9 days. This review shows that the use of customised 3D-printed models is valuable in the education of junior surgeons as well as the enhancement of operative skills for senior surgeons due to a superior visualisation of anatomical networks and pathologic morphology compared to volumetric imaging alone. Furthermore, 3D-printed kidney models may facilitate interdisciplinary communication and decision-making regarding the management of patients undergoing operative treatment for renal disease. It cannot be suggested that a more expensive material constitutes a higher level of user-satisfaction and model accuracy. However, higher costs in the manufacturing of the 3D-printed models reported, on average, a slightly shorter time duration for the 3D-printing process and total manufacturing time.

## 1. Introduction

Three-dimensional (3D) printing in medicine is a rapidly advancing area of research, with applications lying in the orthopaedic and dental industries, as well as recently for the treatment of disease [1,2,3,4]. In recent years, 3D printing has been investigated for its use in creating customised prosthetic implants and medical devices, as well as for pre-surgical rehearsal, mainly in the context of cardiovascular and cerebrovascular diseases, and hepatic and renal tumours [1,2,3,4].

Different studies have explored the feasibility of creating patient-specific, 3D-printed kidney models for applications including education and pre-surgical planning of complex cases of renal disease, due to the ability of 3D models to superiorly encapsulate anatomic spatial relationships [3,5,6,7,8,9,10,11,12,13,14,15,16,17,18,19,20,21,22,23,24,25,26,27,28,29,30,31]. Whilst conventional volumetric imaging modalities such as computed tomography (CT) and magnetic resonance imaging (MRI) are the commonly used imaging modalities for guiding pre-operative planning decisions, they are limited to viewing on a two-dimensional (2D) monitor and therefor lack the ability to demonstrate tumour depth-perception. Furthermore, surgical treatment of renal tumours is moving away from a radical approach, whereby minimally invasive, nephron-sparing approaches are now being utilised where appropriate. This makes pre-surgical planning and confidence in the surgical approach crucial to salvaging the maximum possible amount of healthy tissue. The implementation of 3D-printing techniques in medical practice represents a new approach to facilitate this.

Among the studies which have investigated the value of 3D printing for education and pre-surgical planning of renal disease, there is a discrepancy in findings on what the optimum 3D-printing technology, software, and material(s) are based on factors such as soft-tissue realism, manufacture cost, and time duration. The purpose of this systematic review is to analyse and review current literature on the clinical value and applications of 3D-printed kidney models for renal disease, and to assess the characteristics and costs of different 3D-printing manufacturing tools and materials. It is expected that this systematic review will shed light on current and potential applications of 3D printing for renal disease, and supplement current research in the field of 3D printing in medicine. 

## 2. Methods

This systematic review was performed in accordance with the Preferred Reporting Items for Systematic Reviews and Meta-Analysis (PRISMA) criteria [32]. No ethical approval was required due to the nature of this study.

### 2.1. Search Strategy

A systematic search of the literature was performed utilising five different databases (last search: 31 March 2019). PubMed (including MEDLINE), Scopus, Springer Link, Science Direct, and ProQuest Health & Medical Collection were used to identify relevant and appropriate literature. The keywords and search terms input into each of the databases included “3D printing” OR “three-dimensional printing” OR “3D-printed models” AND “renal disease” OR “renal tumour” OR “renal cancer” OR “renal cell carcinoma”. The keywords were used collectively and in different combinations to generate a comprehensive search.

### 2.2. Inclusion Criteria and Data Extraction

**Figure 1:**
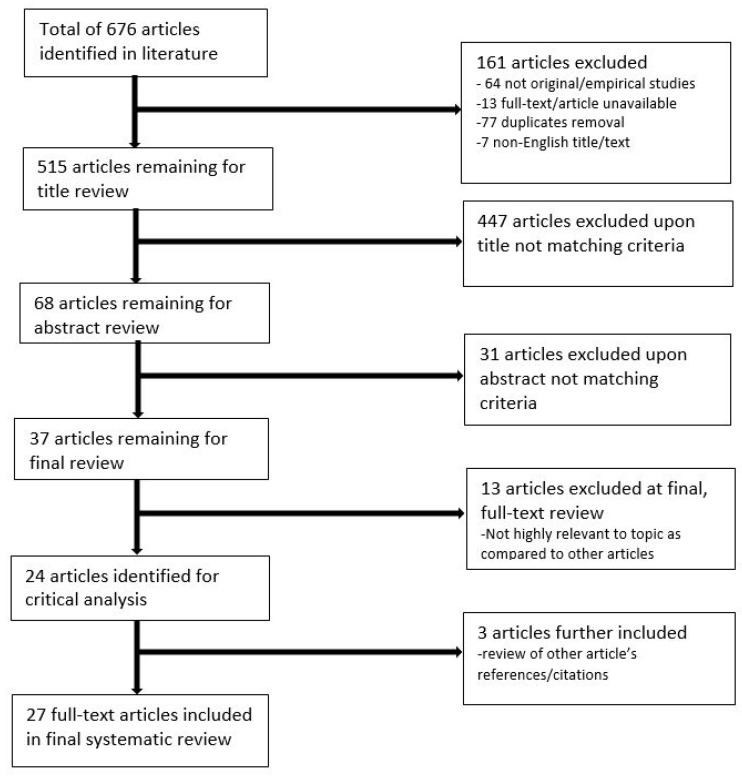
Flow chart demonstrating identification of relevant literature.

Literature included in the review was limited to studies published in the last 10 years (between 2009 and 2019), to ensure only material relevant to current practice was included, and results were limited to English language articles. Studies that were available in full-text, peer-reviewed and original/empirical research (including patient case studies) were included, and editorials were excluded. Articles to be included in the review were screened by two independent reviewers according to the title, abstract, and full-text relevance to 3D-printing applications for renal disease treatment, as well as 3D-printing technology and the associated costs and time durations. At the final stage of the selection process, selected article reference lists were also screened for additional relevant studies to be included. Figure 1 disseminates the synthesis of material retrieved for the systematic review.

## 3. Results

### 3.1. Literature Search Outcomes

Out of a total of 676 articles identified in the original literature search, 24 articles were eligible for analysis, with an additional three articles included from a review of references/citations from other articles. Thus, a total of 27 studies were included in the review (Figure 1). Twenty-five of the studies were original research, and two were case reports. Of these 27 studies, the number of printed 3D models was less than 20 in 26 studies, while the remaining study involved 200 patients who were randomly allocated to either receive pre-operative planning with imaging alone or a combination of imaging and 3D-printed models [14]. 

### 3.2. Study Characteristics

Of 27 studies, 18 utilised CT datasets to construct the 3D-printed models [6,7,9,10,11,12,15,16,17,18,20,21,22,23,24,25,30,31], while five utilised MRI datasets [5,8,19,26,29], and 4 utilised a combination of both imaging modalities [13,14,27,28]. 

Fifty-two percent of studies utilised participants/cases of patients with renal masses highly indicative of renal cell carcinoma (mostly complex), with one case of a patient with bilateral renal tumours [5,6,7,8,9,10,11,14,20,21,25,30,31]. Nineteen percent of studies utilised participants who were eligible or scheduled for undergoing laparoscopic partial nephrectomy surgery [12,13,16,22,24]. Other participants/case types utilised are identified in Table 1, and include patients selected for renal transplantation surgery, or patients selected to undergo surgery for the removal of renal calculi [18,23,27].

### 3.3. Quantitative Analysis of 3D-Printed Kidney Models

Out of the 27 studies reviewed, 22 endorsed a qualitative assessment of 3D-printed kidney models, regarding the usefulness of the models and their applications in areas including pre-operative planning and education [5,6,7,8,9,10,11,12,13,14,15,16,18,20,21,22,24,25,26,27,28,31]. Three studies performed a quantitative assessment of 3D-printed kidney models by comparing them to the original datasets from which they were retrieved [17,29,30], and two studies encompassed both a qualitative and quantitative analysis of 3D-printed kidney models [19,23].

Quantitative analysis of the 3D-printed models was performed by measuring dimensions including width, height and length of anatomy such as the tumour(s) on the 3D-printed models, and comparing values to the original imaging datasets from which the models were retrieved. All studies performing a quantitative analysis of the 3D-printed kidney models found they had a high level of dimensional accuracy compared to the original datasets [17,19,23,29,30]. One study investigating the use of patient-specific, 3D-printed kidney models for simulating renal transplantation procedures also compared the weight of the model to the weight of the donor organ, and found that it only varied by five grams [23]. Different measuring tools such as digital callipers and measuring programs were utilised among studies executing a quantitative analysis of the models, with one study by Adams et al. [17] performing a CT and ultrasound scan on the phantoms and retrieving digital measurements of the resulting data using meshing software. Measurements were achieved by superimposing the CT dataset of the model with the original CT dataset, then selecting three marker points on the kidney and collecting system to measure and compare. Results were such that a small mean error of 0.6 mm was identified among marked points on the kidney, and morphological details of the collecting system read an error of less than 1 mm [17]. The majority of studies calculated the mean difference between measurements of anatomical structures on the model and original dataset, with the study by Adams et al. [17] being the only study to calculate percentage error in different anatomical parts of the model, for which they found a percentage error of 1% across the entirety of the model. Mean differences between measurements ranged from 0.3 to 2.43 mm amongst studies [17,30]. The study conducted by Liu et al. [30] was the only study to perform statistical testing on the measurement values, utilising a t-test to assess for any statistically significant differences between the width of the tumours measured on the models and original CT datasets (*p* > 0.05). The study found no statistically significant variability between measurements for either of the two cases that were printed, suggesting a high degree of model verisimilitude [30]. The study by Woliner-van der Weg et al. [29] investigated the ability of 3D-printed kidney and pancreas phantoms to replicate single photon emission computed tomography (SPECT) characteristics for ^111^In-exendin imaging, and found that the location of artifacts were present in similar locations for all cases, suggesting the phantoms were accurate.

### 3.4. Qualitative Analysis of 3D-Printed Kidney Models

Qualitative assessment of the 3D-printed models was performed in 89% of studies, with most utilising a surveying approach in the form of survey-questionnaires given to participants to evaluate the usefulness and potential applications of the models, as well as the contribution of the models to their knowledge of renal disease [5,6,7,8,9,10,11,12,13,14,15,16,18,19,20,22,23,24,25,26,27,28,31]. Six studies used a rating/scoring system that enabled participants to rate different aspects of the model, such as soft-tissue realism and representation of anatomical networks [10,13,14,18,20,24]. In these studies, ratings were mostly in the form of scores out of 5, 10 or 100, which corresponded to categorical variables such as 1 being very poor/not useful, and 5 being very good/useful. Six studies utilised an in-depth survey-style questionnaire, whereby participant knowledge before and after being presented with the models was evaluated, and questionnaires included open-ended questions rather than a rating system [5,6,9,11,12,31].

Six studies used questionnaires targeted at patient participants with renal disease [6,10,14,18,21,31], with all studies receiving positive responses. A mean of 9/10 and 4.48/5 (*p* < 0.05) was retrieved amongst studies for patient participant rating of the usefulness of the models [6,13,14,18]. These ratings were based on how patients rated their understanding of their disease location, characteristics, and planned surgical treatment utilising the 3D-printed models.

Seven studies used a questionnaire targeted at clinicians practicing in the field of renal disease such as junior medics, registrars, and surgeons [5,9,10,11,12,20,24]. For the studies which used a rating/scoring system, scores ranged from 6.0 to 9.5/10, with a combined mean of 7.44/10 (*p* < 0.05) for the two studies that used a rating system out of 10 [10,24]. Two studies created a questionnaire that assessed which surgical path and procedure surgeon participants would approach upon viewing only imaging datasets, and then again after being presented with the 3D-printed models [5,9]. Results showed that pre-operative decisions were altered slightly after presentation of the models for both studies, with 30–50% and four out of five clinician participants changing their approach to entering the kidney retroperitoneally or transperitoneally for each study, respectively [5,9]. Other studies qualitatively evaluating the use of 3D-printed kidney models assessed clinician participant operative accuracy before and after utilising the 3D-printed models and found that they resulted in an improvement of new and existing technical skills of trainees, and improved clinician anatomical recognition [10,11,12,20,24]. One study by Marconi et al. measured the time spent by clinician participants identifying anatomical structures on a 3D-printed kidney model, a 2D CT dataset of a kidney, and a 3D CT reconstruction of a kidney, and found that the time spent identifying structures on the model was on average 66.37 s shorter than identifying structures on the 2D dataset, and 10.13 s shorter than identifying structures on the 3D reconstruction [12]. The study conducted by Maddox et al. [7] measured intra-operative patient blood loss for clinicians using patient-specific, 3D-printed kidney models as simulation tools prior to performing laparoscopic renal surgery, and compared results with clinicians that did not utilise the 3D models. For the clinicians utilising the models, blood loss was 65% lower (*p* = 0.01) [7].

### 3.5. 3D-Printing Technologies, Materials and Software Tools

**Figure 2:**
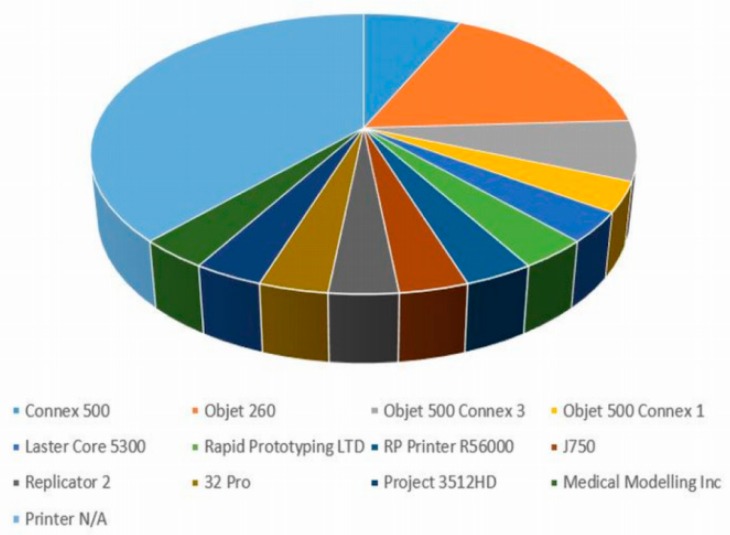
Different 3D-printer models/brands utilised based on the analysis of 27 studies.

Fifteen of the 27 studies provided the type of 3D printer(s) utilised in their studies [5,6,10,11,14,16,17,19,23,24,26,27,29,30,31]. A total of 13 different models/brands of 3D printer were identified amongst the studies reviewed, with the most popular being the commercial 3D printer Objet 260 (Stratasys, Eden Prairie, MN, USA) [17,26,29,30,31] used by 5 studies, followed by the Connex 500 (Stratasys, Eden Prairie, MN, USA) [5,23] and Objet 500 Connex 3 (Stratasys, Eden Prairie, MN, USA) [6,24]. Various other printer brands were utilised by the remaining articles, which are illustrated in Figure 2.

**Figure 3:**
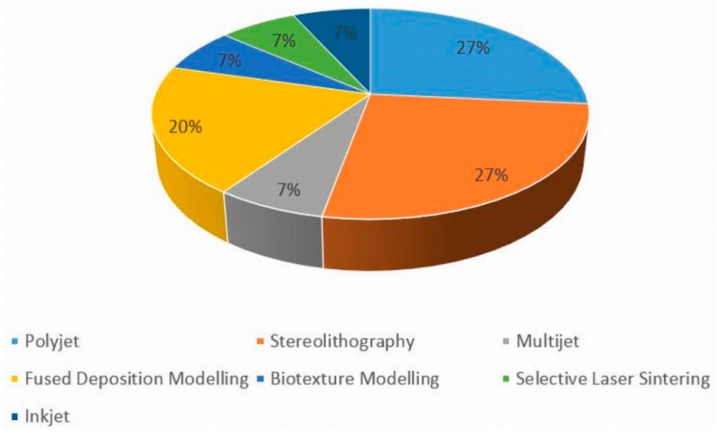
Comparison of different 3D-printing technologies utilised according to the review of these studies.

The most common types of 3D-printing technologies utilised were PolyJet technology [13,27,29,30] and Stereolithography [5,11,20,21], these being encompassed by four studies each. A total of seven different 3D-printing technologies were identified overall amongst the 27 articles reviewed, with the remaining technologies disseminated in Figure 3. Thirteen of the 27 articles did not specify the type of technology utilised [6,8,10,12,14,16,17,19,22,24,25,26,28]. Komai et al. [31] utilised a new technology called Biotexture modelling, whereby multi-material and multi-coloured technologies were implemented for 3D printing the models.

**Figure 4:**
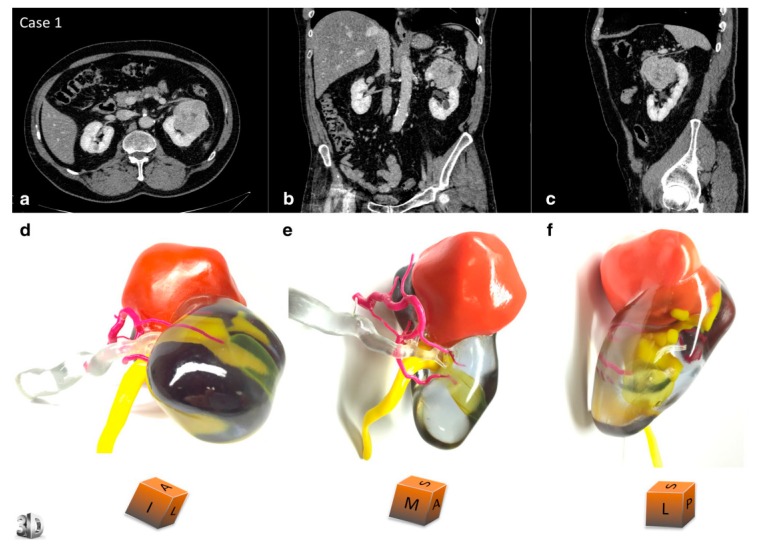
3D-printed model of a 67-year-old male with renal tumour at the upper pole of left kidney. Comparative views of the CT scan at the nephrographic phase ((**a**) axial, (**b**) coronal, and (**c**) sagittal planes) and corresponding views of the physical model ((**d**) superior and median view, (**e**) median and anterior view, and (**f**) lateral view). An inferior polar cyst is also displayed on this model (translucent yellow). The cubes show the 3D-printed model orientation in space (I = inferior face, A = anterior face, L = lateral side, S = superior face, P = posterior face, M = median side). The patient underwent a left radical nephrectomy for a 65 × 56 × 42 mm clear cell renal cell carcinoma, pT1bN0Mx, Fuhrman grade 3. The arterial tree is presented in opaque magenta, the collecting system in opaque yellow, and opaque orange for tumour display. The renal vein and renal parenchyma are kept translucent to allow the best visualisation of the relationships between the renal tumour and surrounding structures. Reprinted with permission from Bernhard et al. [6].

**Figure 5:**
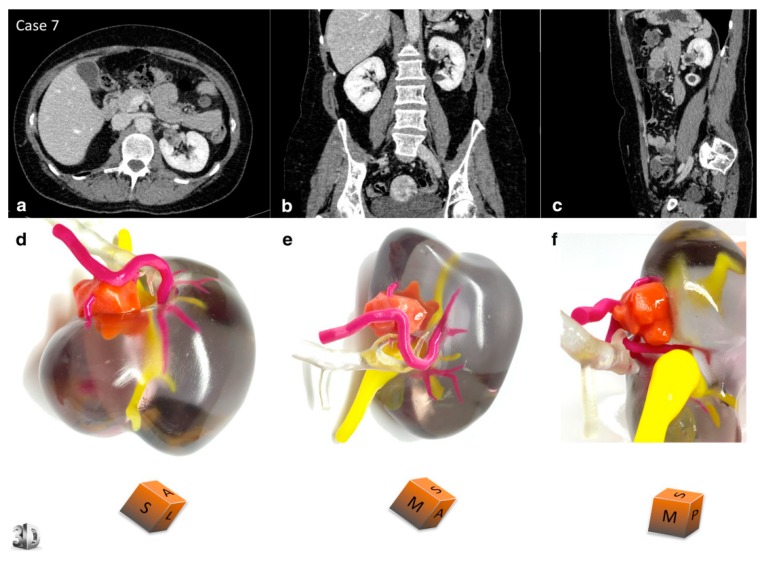
3D-printed model of a 53-year-old female with a renal tumour at the interpolar region of left kidney. Comparative views of the CT scan at the nephrographic phase ((**a**) axial, (**b**) coronal, and (**c**) sagittal planes) and corresponding views of the physical model ((**d**) superior view, (**e**) median view, and (**f**) median view). The cubes show the 3D-printed model orientation in space (I = inferior face, A = anterior face, L = lateral side, S = superior face, P = posterior face, M = median side). The patient underwent a left partial nephrectomy for a 21 × 15 × 15 mm angiomyolipoma. Description of colour corresponding to different renal structures and tumour is the same as in Figure 4. Reprinted with permission from Bernhard et al. [6].

Seventy-eight percent of the articles also detailed the type of material(s) utilised for the construction of the 3D-printed models [5,6,7,8,9,10,13,16,17,18,20,21,22,23,24,26,27,28,29,30,31] (Figure 4 and Figure 5), with the majority of the articles evaluating the properties, advantages, and/or limitations of the material(s) selected. Most of the articles utilised plastic/resin-based materials [5,8,9,20,21], with silicone-based and acrylic-based materials also being popular [22,26,27,28,31]. Four articles evaluated the use of silicone, flexible material in their studies, reporting that it was able to closely mimic the feel and texture of real kidney parenchyma, and provide tear-resistibility and malleability to enable easy cutting in cases of mock surgeries [16,22,26,28]. Photopolymer and acrylic polymer materials were adopted by eight studies, and were also reported to have a high accuracy in being able to replicate the feel of real kidneys and mimic surrounding structures in the abdomen in studies utilising these materials [7,17,20,21,23,24,27,29]. The VeroClear photopolymer brand was employed in three of these studies [17,23,29]. Other higher quality materials identified include TangoPlus, Polylactic acid filament, and thermoplastic polymer material(s), as displayed in Table 1 [10,13,16,20,23,30]. A study by Liu et al. [30] compared a 3D-printed kidney model manufactured using a commercial 3D printer to a model manufactured with a homemade printer, with differences found in the material used for 3D printing. TangoPlus material was utilised for the model printed commercially, whilst Polylactide material was used for the model manufactured using the homemade printer. The study reported that whilst model accuracy was similar, the 3D-printed model manufactured using the homemade printer showed a slightly inferior demonstration of renal calyces from an external view compared with the commercially manufactured model, despite a similar demonstration from an internal view. The study also suggested that there is greater potential for multi-coloured printing using a commercial 3D printer compared to a homemade one [30].

The majority of the studies provided the type of post-processing and segmentation software tools/programmes that were utilised, with the Mimics (Materialise, Leuven, BE) versions 14.0, 16.0, 18.0, and 20.0 software for segmentation utilized by six studies [5,11,14,18,27,29]. An array of other segmentation software tools were also used amongst the studies, such as Analyze12.0 and 3D Slicer [16,19,30], displayed in Table 1. The most common post-processing software used was Blender (Blender Foundation, Amsterdam, The Netherlands), which was utilised by four out of 27 articles [9,24,26,28]. Many of the studies also utilised computer-aided design (CAD) software such as TinkerCAD or SolidWorks for post-processing, and Meshlab and Meshmixer were also frequently employed software [9,16,19,23,24,26,27,28,29,31]. Six of the 27 reviewed studies did not specify the type of software/program(s) used for image segmentation and post-processing.

### 3.6. Manufacturing Cost and Time Duration

The cost of 3D printing the models was discerned in 52% of the articles, ranging from USD 1 to 1000 per model [5,6,9,10,12,15,16,17,18,22,24,26,30,31]. The study by Liu et al. [30] compared the cost of 3D printing using a commercial 3D printer to a homemade one, with reported costs being USD 200 commercially compared to USD 1 homemade, with the homemade printer providing kidney models of similar accuracy to the commercial printer, at a much lower cost. Forty-three percent of the studies that stated cost reported costs in the range of USD 100–200 [9,10,12,16,18,30]. Forty-eight percent of the reviewed literature reported the time taken for 3D printing, with duration ranging vastly between 15 min [26] and 3–4 days per model [10]. The recent study by Liu et al. [30] juxtaposed the time taken for 3D printing using a commercial vs. homemade printer, taking 3.5 h commercially as opposed to slightly longer at 4–6 h homemade (per model). Only four of the 27 studies stated the time duration for the segmentation and post-processing of the 3D-printed models [5,22,27,30]. Time durations ranged from 1.5 h to 7 h, with two of the four studies reporting an approximate 1.5 h time duration for the segmentation and post-processing of each kidney model [20,22]. 

The time duration reported for studies that utilised the Connex 500 3D printer ranged from 4 to 9 h, with costs ranging from USD 450–1000 per model. The studies that employed the Objet 260 3D printer stipulated lower costs of USD 200–650 per model, with a longer time duration than the Connex 500 models of up to 25 h. Other, less widely used 3D printers, such as the Laser Core 5300 and Replicator 2 printers, reported lower costs and longer time durations of USD 150 and 4 days, and USD 120 and 4 h 55 min, respectively. The study by Dwivedi et al. [19] investigated the feasibility of 3D printing six tumour moulds and a single kidney model with an advanced-grade tumour, utilised a 3D printer called the Projet 3512HD to print kidney moulds and was the only study which found a large range of costs in the 3D-printing process using the same printer. The study reported a range of USD 20.9–350.7 per 3D-printed tumour mould, and a cost of USD 1000 for the kidney and advanced-grade tumour model. The study also reported a time duration of 12–14 h per tumour mould. Whilst no causal relationship was identified, studies that reported higher costs in the manufacturing of the 3D-printed models also reported, on average, a shorter time duration for the 3D-printing process and total manufacturing time.

### 3.7. Study Purpose

The majority of the studies reviewed (59%) investigated the clinical value and application(s) of 3D-printed kidney models for pre-operative planning and intra-operative simulation of renal disease [5,7,8,9,10,13,14,15,16,17,22,23,25,27,28,31]. Thirty percent of the studies evaluated the usefulness of 3D-printed kidney models for the education of junior and/or senior clinicians in their understanding of renal anatomy and renal pathology [9,11,12,16,20,21,24,26]. Twenty-two percent of the studies investigated the application(s) of 3D-printed models for patient awareness and education of their renal condition, such as the understanding of basic kidney anatomy, the location and characteristics of their renal disease, and the nature of the surgery they would undergo [6,10,14,18,21,27]. Twenty-two percent of studies also investigated one or more of the above. Four of the 27 articles exhibited study purposes lying outside patient/clinician education and pre-surgical planning. Other study purposes are depicted in Table 1 and include comparing the accuracy of 3D-printed models of diseased kidneys printed from commercial versus homemade printers [30], the implementation of 3D-printed kidney models to facilitate interdisciplinary communication for treatment and management [15], and the application of 3D-printed kidney models in other imaging/surgical modalities such as radiomics [19,29].

## 4. Discussion

### 4.1. Key Findings

This critical review analyses 27 studies that investigated the clinical value and applications of 3D-printed kidney models for renal disease. Based on the analysed literature, it can be discerned that 3D-printed kidney models can be successfully generated from CT and MRI imaging with a high level of anatomic accuracy. Furthermore, 3D-printed kidney models are a useful resource for pre-surgical planning and surgical rehearsal for complex cases, with benefits including reducing estimated blood loss and reduced chances of intra-operative complications occurring [5,7,8,15,16,18,23,25,26,27,28,31]. Three-dimensional-printed kidney models are also a useful tool for patient education on their condition, and can be a medium to enhance patient-practitioner interaction [6,10,14,18,21,27]. Out of the eight studies evaluating patient participant perception of their personalised 3D-printed models, all reported high patient satisfaction levels [6,10,13,14,18,20,21,31]. It was elucidated that patient participants found the 3D-printed models valuable for improving their comprehension of the disease, and that the models were a useful tool to guide collaboration between the patient and surgeon in deciding the best treatment approach. The study by Silberstein et al. [21] also included family members of the patient as participants, and reported a high level of satisfaction, suggesting that family members felt more educated on the condition of their loved one after being presented with the model. The inclusion of family members was advantageous as it provided an additional perspective on the value of 3D-printed kidney models that was not considered by other studies. Results also show that 3D-printed kidney models may be valuable assets in areas such as clinician education (e.g., the education of junior doctors on disease morphology), and can also facilitate interdisciplinary communication between different specialists in regards to disease treatment decisions [9,11,12,15,16,20,21,24,26]. In addition to evaluating 3D printing in the context of kidney disease, four studies also evaluated 3D printing for diseases in other organs such as the pancreas, prostate, and spleen [12,13,14,29]. The main findings of these studies suggest that 3D-printed models may be useful for the surgical treatment, management, and education of disease in these other organs. Through qualitative and quantitative analysis of findings on the clinical value of 3D-printed kidney models, this review suggests that 3D-printed models have a promising role in the areas of education and treatment.

A similar systematic review that analysed 15 studies on 3D printing for renal disease was also published recently [33]. The current review is different, in that it includes a detailed analysis of 27 studies, and aims to further advance the previous research by investigating different 3D-printing technologies and printer types, as well as segmentation and post-processing software and their impact on the 3D-printing process. In addition to previous research, this review discusses a greater array of 3D-printing applications, characteristics, and limitations, and it recommends potential applications for 3D-printed renal models based on the latest literature. 

### 4.2. Participant Types

For the generation of 3D-printed kidney models, 14 studies utilised patient participants who had renal masses, and five studies utilised patient participants who were selected for partial nephrectomy surgery. Twenty-six of the 27 studies used cases based on real patients, with the remaining one utilising three kidney cadavers to create the 3D-printed models [17]. The two case studies included in this review utilised complex cases that stood out and inherently demonstrated the clinical value of 3D-printed models for aiding difficult cases. The case study conducted by Libby et al. utilised the case of a patient who had a tumour that extended into the adrenal gland and inferior vena cava, and evaluated the usefulness of 3D-printed kidney models for performing a mock surgery on such a case [8]. The study found that the 3D-printed model enabled a superior visualisation of the tumour thrombus and how it was situated in relation to healthy anatomy, and consequently improved surgeon confidence in the operative technique [8]. The case study by Golab et al. [15] utilised the case of a patient with a giant renal tumour reaching the venous system and right atrium, and the study results were similar in that viewing the 3D-printed kidney models prior to surgery led to improvement in intra-operative safety and reduced operative times. The study conducted by Atalay et al. [18] utilised the cases of five participants who were to undergo surgery for unilateral staghorn calculi, with the 3D-printed renal collecting system printed in the models. In this study, the 3D-printed models provided a superior enhancement of the calculus location, and therefore facilitated decisions regarding the surgical approach to be taken for calculus removal [18]. The studies utilising complex patient cases concluded that the implementation of 3D-printed kidney models for the education and pre-operative planning of rare cases and surgical procedures that are not commonly performed could markedly improve treatment outcomes for these patients.

### 4.3. Clinical Value and Applications in Pre-Surgical Planning

As discussed earlier, 59% of the studies reported the clinical value of 3D-printed kidney models largely in the field of pre-surgical planning. Findings captured by all of these studies were positive in regards to this clinical application. This was a result of the ability of the 3D-printed model, when printed with a high degree of verisimilitude and structural accuracy, to capture the internal and external complexity of the organ to scale. All studies performing a quantitative analysis of the 3D-printed models found that anatomical dimensions measured very similarly for both the 3D-printed models and original CT datasets, suggesting that the models had a high degree of accuracy. Many of the reviewed studies reported that this ability enabled clinicians to have an enhanced demonstration of the internal dimensions of the patient’s kidney prior to performing surgery, and even carry out a rehearsal surgery. Despite the 3D reconstructions available with volumetric CT and MRI imaging, the depiction of a 3D, complex organ on a 2D monitor is a limitation that can be overcome with the use of a 3D-printed model, showing features such as tumour depth and extent more vividly than traditional visualisations.

Eighty-one percent of the studies assessing the clinical value of the models for pre-surgical planning reported that the 3D-printed models facilitated pre-operative planning decisions, especially in cases of minimally invasive, nephron-sparing surgeries [5,6,7,8,9,10,11,12,13,14,15,16,17,18,19,20,23,24,25,26,27,28]. Two studies reported that utilising the 3D-printed kidney models as simulation tools impacted decisions regarding the surgical access point and even resulted in a slight deviation of the original surgical path [5,9]. The study by Monda et al. [26] investigated the ability of silicone-based 3D-printed kidney models for use as educational resources for trainee surgeons, and found that the models were highly useful in improving technical cutting skills of trainees, as well as being a valuable resource for practice and learning. This was also due to the models providing a high degree of organ-realism and being easy to cut. Two studies reported that implementing the models has the potential to reduce intra-operative renal ischemia times and improve surgical-efficiency, due to increased confidence gained by exposure to the kidney via simulation prior to live surgery [7,16]. The study by Maddox et al. [7] also reported a significant reduction in estimated intra-operative blood loss, due to the ability to become familiar with the patient’s anatomy and establish any deviations from the planned approach prior to surgery. Whilst phantoms present many pre-operative and intra-operative benefits, the only limitation identified among the studies is that pre-surgical rehearsal may be unfeasible if there is a limited amount of time available prior to surgery [27]. While it was not specified by any of the studies under review, the use of 3D-printed models for pre-operative planning may also enable the salvaging of a greater amount of tissue intra-operatively, which is crucial for small-scale surgeries such as nephron-sparing or laparoscopy; however, this is an area that remains open to further investigation.

### 4.4. 3D-Printing Technologies and Materials

An analysis of the materials utilised for 3D printing indicates that silicone-based and acrylic polymer/photopolymer material(s) such as TangoPlus are the most useful for replicating kidney tissue realism. Many studies also utilised coloured materials or manually coloured the material to create a more visually accurate 3D-printed model. Colours such as reds, blues, yellows, and pinks were commonly used to mimic parenchyma, vasculature, and the tumour or diseased area (Figure 4 and Figure 5). Five studies also used clear, translucent plastic/resin to craft the renal parenchyma, and only coloured the diseased area/tumour, which was reported to assist in identifying spatial relationships between the tumour and healthy structures [6,20,21,23,24,29]. The study by Adams et al. [17] was the only study that performed a quantitative analysis of the material properties of the three 3D-printed models—all of which were manufactured from a different material—then compared them to real kidney tissue. This was achieved by measuring the tensile strength and elastic modulus of the chosen material to that of a real kidney. The study found that silicone-based material has a modulus that closely resembles real kidney tissue. Material acoustic properties were also measured using ultrasound, whereby the Agarose gel model had the closest echogenicity to a real kidney compared to the other 3D-printed models [17]. A study conducted by Monda et al. [26] critiqued the use of silicone-based materials for 3D printing, suggesting that the silicone used was not able to offer sufficient soft-tissue realism. The study also recommended further research regarding an improvement in the material(s) used, focusing on achieving a texture and malleability that is less synthetic and more organ-like [26]. This contradicts the findings found by Adams et al. [17], who reported that silicone had material properties closely resembling kidney tissue. The discrepancy in findings suggests that material feasibility may be user-dependent, and may be an area requiring further research in order to determine the optimal material(s) for kidney models.

There were a range of different 3D-printer models and 3D-printing technologies utilised amongst the 27 reviewed studies, suggesting that material availability, material compatibility with printing technology, and user-preference are all influencing factors. The studies that used the Objet 260 and Connex 500 printers utilised more expensive, branded materials such as PolyJet technology and TangoPlus material, while materials such as photopolymers, polylactides, and thermoplastics were used in studies that utilised other, less expensive printers. It was also found that the studies that utilised more-expensive printing technologies used multi-coloured materials, in order to superiorly differentiate diseased from healthy tissue on the model. However, there were some studies using cheaper materials that also used colour, such as the study by Zhang et al. [10], which used thermoplastic material and performed manual colouring of the renal parenchyma, tumour, and vasculature to improve the demonstration of anatomical networks. Three-dimensional printed models were found to have a high level of patient and clinician satisfaction and anatomic accuracy across all reviewed studies, thus it cannot be suggested that a more expensive material constitutes a higher level of user-satisfaction and model accuracy. Therefore, whilst it is unlikely that a material costing USD 1 could possess a superior level of soft-tissue realism to a material costing USD 1000, positive findings were found in the light of all studies regarding the usefulness of 3D-printed models, and different studies demonstrated different material preferences. There were no notable differences in findings presented by the studies that utilised more expensive technologies and materials compared with studies that utilised cheaper technologies and materials to suggest that no gold standard 3D-printing technology could be identified by this review. 

### 4.5. Limitations

An analysis of the literature selected for review suggests that whilst there are many advantages provided by 3D-printed kidney models, several limitations surrounding the cost and time of manufacturing exist. The first limitation identified from the 27 articles analysed is that a considerable amount of time is required for data segmentation and post-processing in order for the 3D-printing process to be successful and accurate [1,2]. The segmentation and post-processing stage can take up to 7 h, as shown in the study by Wake et al. [5], even with the aid of automatic and semi-automatic processing tools, and the 3D-printing process can take up to 4 days per model, as demonstrated in the study by Zhang et al. [10]. Thus, despite the clinical value and vast applications of implementing 3D-printed models into the treatment of renal disease, it is impeded by manufacturing time and requires further research to overcome this limitation. 

A second limitation encountered by the studies is that multiple software tools are required for segmentation and/or post-processing, with 10 of the 27 articles reporting the use of more than one program or software. This may, in turn, incur further costs to download. Furthermore, the overall process from where the data is segmented until the models can become useable also has a long time duration, with the study by Komai et al. [31] reporting a duration of up to 9 days for the overall manufacturing process of the models.

The cost of 3D printing is a main limitation identified that impedes the implementation of 3D printing in routine practice, with costs being up to USD 1000 per kidney model for printing with high-quality TangoPlus and PolyJet materials [5,10,13,16,20,23,30]. Whilst other, lower-quality materials and printing technologies can be implemented for costs ranging between USD 100 and 200, it may be at the expense of accuracy of the renal tissue texture, and may correspond with an increase in manufacturing time duration. [9,10,12,16,18,30]. The study by Liu et al. [30] reports a cost as low as USD 1 for 3D printing using a homemade 3D printer; however, the time duration was longer at 4–6 h, compared to 3.5 h using a commercial printer. The study also reports that there was little opportunity to print in multiple colours using a homemade printer, due to the associated cost and materials required, which may be a limitation, as using different-coloured materials is effective in the differentiation of different tissues and structures. As the costs reported by the analysed studies are quite variable and technology and material-dependent, further research is needed to find a solution to mitigate costs of 3D printing in medicine.

There are also limitations regarding this critical review. Whilst most of the studies evaluate the 3D-printed models from a qualitative perspective, only five of the 27 reviewed studies performed a quantitative assessment of 3D-printed kidney models [17,19,23,29,30], and only two of these five studies performed a quantitative analysis utilising more than a comparison of the mean tumour dimensions [17,30]. Additionally, only one study performed a statistical analysis of the measurements [30]. Hence, in order to provide more evidence on the ability of models to replicate kidney anatomy and pathology, other anatomical structures should also be measured and compared, and more literature encompassing an in-depth quantitative analysis is required. Furthermore, multiple studies failed to provide information on the type of software used for data segmentation and post-processing, with 23 out of 27 studies also failing to report the time duration for this process. Therefore, limited information was generated on the segmentation and post-processing processes, with very limited evidence on the time duration required for this step. Additionally, as the area of 3D printing for the treatment of kidney disease and renal cell carcinoma is a growing area of research, there were only a limited amount of relevant studies available for review, with a total of only 27 of the most relevant being selected for critical analysis. In the majority of these studies, only a small number of patients/cases were selected, due to the high costs associated with 3D printing. Further research in this field with a larger sample size and more information on the segmentation and post-processing step is required to generate a more robust analysis of the feasibility of 3D-printed kidney models for implementation into medical education and treatment of renal disease. More robust studies such as randomised controlled trials (RCTs) are still lacking in this area when compared to 3D printing in heart disease, with several RCTs available in the literature [34,35,36].

## 5. Conclusion and Future Recommendations

The purpose of this systematic review was to evaluate the clinical value and applications of personalised, 3D-printed kidney models for pre-surgical planning and education of renal disease, as well as to investigate the software tools and technologies suitable for 3D printing. The key findings of the literature are such that 3D-printed, patient-specific kidney models are able to accurately represent 3D spatial relationships between different anatomical and pathological structures and present as useful resources for pre-surgical planning and simulation of surgery for renal disease, especially in complex and rare cases. Furthermore, the literature reveals that the use of customised 3D-printed models is valuable for the education of junior surgeons, as well as for the enhancement of operative skills for senior surgeons, due to a superior visualisation of anatomical networks and pathologic morphology compared to volumetric imaging alone. In addition to this, the literature has suggested that 3D-printed kidney models may facilitate interdisciplinary communication and decision-making regarding the management of patients undergoing operative treatment for renal disease, as they are a mechanism that can be understood by a range of professionals, including those that are not familiar with volumetric images as part of their occupation. The literature findings also indicate that 3D-printed kidney models can educate patients and their families on understanding the surgical process as well as the characteristics of their disease. It has been revealed that a range of 3D-printing technologies, printer models, and materials exist, and that no gold standard has been identified, as it is based on user-preference. Despite this, study findings suggest that the utilisation of different colours may aid in separating healthy from diseased renal tissue, further benefitting the pre-surgical planning process.

The reviewed literature stipulates that while 3D-printed kidney models may have multiple applications in medicine, prevalent limitations such as the high costs and lengthy time durations for the 3D printing and manufacturing processes, which require further investigation in order to improve accessibility to 3D printing in a clinical context. Further research focusing on these areas and encompassing a larger sample size with in-depth quantitative assessment may be able to strengthen findings reported on the accuracy and feasibility of 3D-printed kidney models for the treatment and education of renal disease. An analysis of the literature has demonstrated that the diagnostic and treatment process of renal disease may be assisted if surgeons are able to carry out a mock surgery using models beforehand.

## Figures and Tables

**Table 1:** Summary of findings of these 27 studies that were reviewed.

First Author and Publication Date	Study Sample Size and Participants	Study Purpose	Technology/Software Used for Segmentation and Post-Processing/Time Duration	Imaging Modality Used for 3D Printing	3D Printing Technology/Material/Costs/Time Duration	Key Findings
Wake et al. 2017 [5]	10 renal mass cases collected. 3 urologists used as participants for qualitative questionnaire.	Assess ability of patient-specific 3D-printed kidney models with tumours to enhance pre-surgical planning for complex cases of RCC.	Mimics 16.0 (Mimics, Materialise, Leuven, BE) software. 7 h time duration.	MRI	Flexible, transparent material (HeartPrint Flex, Materialise, Leuven, BE) used with cyan and magenta rigid material combinations (VeroCyan and VeroMagenta, Stratasys, Eden, Prairie). Stereolithography technology using Connex 500 (Stratasys, USA) 3D printer. USD 1000 per kidney model. 10 h taken per model.	High degree of verisimilitude and good correlation between tumour size measurements of 2D data and 3D model. 3D models impacted clinician pre-surgical planning decisions, most specifically in the trans-peritoneal or retroperitoneal approach and clamping. 3D models could be valuable for pre-surgical planning of complex cases and reduce occurrence of intra-operative complications.
Bernhard et al. 2016 [6]	7 patients with primary renal tumours of sizes 2.5–7.2 cm.	Investigate ability of 3D-printed models of kidneys with renal tumours to facilitate patients’ understanding and education of their condition.	Image recognition algorithm Synapse 3D (Fujifilm, Tokyo, Japan. Time duration N/A.	CT	Combination of opaque magenta, opaque yellow, and transparent photopolymer materials. Objet 500 Connex 3 (Stratasys, Eden Prairie, MN, USA) 3D printer. USD 560 per kidney model.	Significant improvement in patient’s understanding of their planned surgical procedure, and kidney physiology and anatomy after 3D model presentation (37.6% improvement, *p* < 0.05). Patient satisfaction with the usefulness of the models and their experience was on average 9.4/10.
Maddox et al. 2018 [7]	6 patients with 7 enhancing tumours (1 bilateral) ranging from 2.8–5.5 cm.	Investigate the feasibility of using 3D-printed kidney models for pre-operative simulation of renal tumour resection and application into simulation labs.	3D Systems (Rock Hill, SC). Time duration N/A.	CT	Photopolymer, flexible resin material (translucent for parenchyma and red for tumour). Multi-jet 3D printer (brand N/A). Time-duration and cost N/A.	3D models were able to closely resemble the feel and texture of real kidneys and thus assisted in the education of junior urologists and their anatomical understanding. Considerably lower estimated blood loss in patients with pre-surgical simulation using the 3D models prior to surgery (*p* = 0.01).
Libby et al. 2017 [8]	1 case of a 76-year-old woman with right mass extending into adrenal gland and inferior vena cava.	Assess ability of 3D-printed kidney models to guide surgical treatment of complex cases of renal disease.	Software and time duration N/A.	MRI	Deep red colour for thrombus and pink colour for parenchyma and vasculature. 3D printer, time duration and cost N/A.	3D-printed model was able to improve knowledge of the patient and patient’s family on their condition, required treatment, and general kidney anatomy. 3D-printed kidney model improved visualisation of tumour thrombus and its relationship to surrounding critical anatomy, promoting surgeon confidence in the interventional technique.
Glybochko et al. 2018 [9]	5 patients with renal tumours.	Assess usefulness and clinical applications of soft, 3D-printed kidney models for localised surgical treatment planning of renal disease.	Amira Version 5.4.4 (license ASTND.44644) software; Meshmixer (Autodesk, Inc., San Rafael, CA, USA); Blender (Blender Foundation, Amsterdam, the Netherlands, open-source software). Time duration N/A.	CT	Red, blue, and yellow polylactic acid plastic material Fused Deposition Modelling technology (Cura, open-source software). 3D printer brand N/A. Printing duration of 10–20 h per kidney model and total model completion duration of 2–4 days. USD 150–450 per mode.	3D-printed model analysis resulted in a change in initial surgical approach and access method (3/5 surgeons changed their approach to partial/radical nephrectomy surgery and 4 surgeons changed their initial decision for transperitoneal/retroperitoneal access). 3D-printed models demonstrated anatomical structures and tumour location superiorly, and thus are useful tools for pre-operative training and trainee education.
Zhang et al. 2016 [10]	10 patients with solitary renal tumours clinically indicated for laparoscopic partial nephrectomy.	Investigate the value of 3D-printed models for laparoscopic partial nephrectomy planning, surgical training, and patient education on their condition and management.	Medical Imaging ToolKit (MITK) and 3DMed. Time duration N/A.	CT	Thermoplastic plastic material with manual colouring of vasculature, parenchyma, collecting system, and tumour performed. LaserCore5300 (Longyuan Rapid Prototyping LTD., Beijing, China) 3D printer. USD 150 per model. 3–4 day duration per model.	The overall usefulness of the models, impact on pre-surgical planning and training, and verisimilitude to real kidneys were rated as 7.8, 6.0, and 7.3 out of 10, respectively. Patients were satisfied with the usefulness of the models (9 or over out of 10 in all questions).
Yang et al. 2018 [11]	1 case of a kidney with a retroperitoneal tumour. 30 participants for model evaluation (10 students, 10 surgeons, 10 residents).	Investigate usefulness of 3D-printed kidney models in enhancing understanding of retroperitoneal tumour anatomy and surgical procedure.	Mimics v.14.01 (Materialize Corp, Leuven, Belgium) software. Time duration N/A.	CT	Stereolithography RP printer RS6000 (Shanghai Union 3D Technology Corp., Shanghai, China). Time duration and cost N/A.	Junior surgeon participant success of anatomical recognition and identifying correct vasculature was improved at 83.33 on 3D-printed model compared to 73.33 on 3D imaging (out of 100). 3D-printed model demonstrated little advantage over 3D imaging for the surgeon participants.
Marconi et al. 2017 [12]	15 patients scheduled for laparoscopic splenectomy, nephrectomy and/or pancreatectomy (2 patients with renal tumours). 30 participants for model evaluation (10 medical students, 10 general surgeons, 10 radiologists).	Assess if 3D-printed models can provide a superior anatomical representation to standard 2D and 3D visualisations for pre-surgical planning of renal disease and surgical training.	Visualisation software Paraview (http://www.paraview.org). Time duration N/A.	CT	Technology and printer N/A. USD 150–200 per model. 20–30 h taken per process.	The highest percentage of correct answers in identifying anatomical structures was achieved for the 3D-printed model group (53.9%), compared to 3D reconstructions (53.4%) and 2D images (45.5%). There was less time spent by participants evaluating anatomy on the 3D models (60.67 ± 25.5 s) compared to 3D reconstructions (70.8 ± 28.18 s) and 2D images (127.04 ± 35.91 s).
Porpiglia et al. 2017 [13]	18 patients (8 undergoing robot-assisted radical prostatectomy, 10 undergoing minimally invasive partial nephrectomy).	Clinical value of 3D-printed models for pre-surgical planning of robot-assisted prostate cancer and nephron-sparing surgeries, training, and education of both urologists and patients with the condition.	Software M3DICS. Time duration N/A.	CT (renal tumour cases) MRI (prostate cancer cases)	Selective laser sintering with thermoplastic polymer technology was used for the kidney models, and PolyJet technology was chosen for the prostate models. Printer brand, time duration and cost N/A.	Patient participants rated the 3D-printed models on average 10/10 for their educational benefits and ability to improve comprehension of the disease. The usefulness of the 3D-printed kidney models for pre-operative planning and comprehending surgical complexity was rated 8/10 by participants.
Wake et al. 2019 [14]	200 patients with confirmed prostate cancer OR RCC to undergo surgical treatment (151 prostate cancer, 49 kidney cancer).	Investigate the value of using augmented reality or 3D-printed kidney and prostate models for patient education on their condition, as well as pre-surgical planning for renal and prostate cancer.	Mimics 20.0 (Materialise, Leuven, Belgium). Time duration N/A.	MRI and CT	Multi-coloured 3D printing (J750, Stratasys, Eden Prairie, MN). Time duration and cost N/A.	Patients described having a better understanding of their disease, tumour characteristics, location, and surgical procedure/plan utilising the 3D-printed models compared to augmented reality models and volumetric imaging.
Golab et al. 2016 [15]	1 patient with a giant renal tumour and neoplastic mass reaching right atrium and venous system.	Assess implementation of 3D-printed models into pre-surgical planning for a rare, complex surgery requiring interdisciplinary communication.	Software and time duration N/A.	CT	Fused Deposition Modelling technology (printer brand N/A). Euro 100/USD 123 per model. 22 h time duration per model.	3D-printed models improved interdisciplinary communication between physicians of different specialisations, facilitating treatment decisions regarding renal disease. 3D-printed models aided pre-surgical planning and visualisation of anatomy, reducing surgery duration and improving surgical safety.
Golab et al. 2017 [16]	3 patients with renal tumours eligible for partial nephrectomy surgery.	Evaluate the clinical value of 3D-printed kidney models for training purposes in the context of laparoscopic partial nephrectomy surgery.	3D Slicer (Surgical Planning Lab, Harvard University, MA, USA) program; TinkerCAD (Autodesk, San Rafal, CA, USA) software. Time duration N/A.	CT	Polylactic acid filament material Elite Double 8 (Zhermack Spa, Italy) silicone material for parenchyma Replicator 2 (MakerBot Industries, LLC) 3D printer 120 USD per kidney model Kidney and tumour form printing duration of 4 h 15 min and 55 min, respectively. Total time duration of 7–8 h per kidney model	The accuracy of the models and silicone flexible material closely mimicked real kidney tissue and enabled easy cutting of the model for surgical simulation. The models improved trainee recognition of renal structures and tumour anatomy and hence the surgical process. Implementation of patient-specific 3D-printed kidney models may assist in shortening laparoscopic surgery times and intra-operative renal ischemia.
Adams et al. 2017 [17]	3D-printed kidney models based on 3 kidney cadavers of persons over 18 years. 10 kidney phantoms with 3D-printed collecting systems.	Assess the ability of 3D-printed kidney models to simulate endoscopic urological procedures, and thus assist in pre-operative planning.	InVesalius 3.0.0 (Centro de Technologia da Informacao Renato Archer, Brazil). Models scaled down 80% in all dimensions for printing. Time duration N/A.	CT	Collecting system was printed using an engineered wax material UV curable photopolymer (VeroClear) material used for outer moulds 3Z pro (Solidscape, NH, USA) 3D printer used for collecting system; Objet 260 (Connex, Stratasys, Israel) 3D printer for outer moulds. 25 h taken for 3D printing of engineered wax material and 4.3 h for 3D printing of VeroClear material. Total of 2 days taken for model to become useable phantom. Cost N/A.	The three 3D-printed models accurately resembled morphological details of the renal collecting system and anatomy (0.6 mm distance error for phantoms). 3D-printed collecting systems were able to be easily visualised endoscopically, promoting possibilities for implementation into endoscopic training.
Atalay et al. 2017 [18]	5 patients with unilateral staghorn renal stones and indication of percutaneous nephrolithotomy.	Assess if personalised 3D-printed kidney models can improve patient understanding of their condition prior to undergoing pelvicalyceal surgery.	Mimics 16.0 (Materialise, Belgium). Time duration N/A.	CT	Fused deposition modelling technology, using a polymer filament (Stratasys Inc.). Printer model N/A. 100 USD per model. 2 h taken per model.	3D-printed models were able to assist pre-surgical planning for collecting system access in complex renal stone-removal cases. Physician-patient interaction and communication was improved after the presentation of customised 3D-printed kidney models (patients rated a 50% improvement in conversation). Patient understanding of basic kidney anatomy and the location of their renal calculus and planned surgical approach improved by 50% (*p* < 0.05).
Dwivedi et al. 2018 [19]	6 patients with renal tumours > 2.5 cm and eGFR <0.30 mL/min/1.73 m^2^.	Assess usefulness of patient-specific 3D-printed kidney moulds for radiomics and radiogenomic analyses.	3D slicer (http://www.slicer.org); CAD software SolidWorks (Dassault Systemes, Velizy-Villacoublay, France). Time duration N/A.	MRI	Project 3512HD (3D Systems, Rock Hall, SC) 3D printer. USD 160.7 ± 111.1 per mould (range USD 20.9–350.7). 1 patient had an entire kidney and large tumour printed for USD 1000. 12–14 h taken per mould.	Study is the first report of patient-specific 3D-printed renal models to correlate MRI imaging features with kidney tumour histopathology. MRI patient-specific 3D moulds of renal tumours are able to facilitate tissue-based analyses for radiomics and radiogenomic studies.
Knoedler et al. 2015 [20]	6 cases of kidneys with renal tumours.	Investigate the effects of 3D-printed renal models with enhancing masses on junior medics’ understandings, localisations, and characterisations of renal tumours.	Software and time duration N/A.	CT	Translucent plastic resin material. Stereolithography (SLA) (3D printer brand N/A). Cost and time duration N/A.	3D-printed models improved trainee nephrectomy accuracy rating significantly compared to CT images (*p* < 0.01). Implementation of 3D-printed kidney models can improve junior medical understanding and characterisation of renal pathology.
Silberstein et al. 2014 [21]	5 cases of kidneys with suspicious renal tumours.	Disseminate impact of personalised, 3- printed kidney models with enhancing lesions on education of trainees, patients, and surgeons for characterisation and management of RCC.	Software and time duration N/A.	CT	Translucent resin for parenchyma and red translucent resin for lesions and proximal ureter. Stereolithography printing technology (Medical Modeling Inc., Golden, CO.). Printer brand N/A. Time duration and cost N/A.	Medical trainees showed improvement in their understanding of tumour characteristics and anatomical relationships. Patients and patients’ families suggested an improvement in their comprehension of their condition and the location and size of the tumour, alongside the planned surgery.
Smektala et al. 2016 [22]	5 patients undergoing lapraroscopic kidney tumorectomy for RCC.	Investigate feasibility of implementing low-cost, customised silicone kidney replicas for pre-operative planning and simulation of complex nephron-sparing surgeries.	94 min time duration per kidney model. Software N/A.	CT	Silicone material. 14 h and 30 min per kidney model. USD 7.4–14.4 per kidney model. Printing technology/printer brand N/A.	3D printing of the models was simple to perform and inexpensive, and thus may be feasible to implement into practice. Silicone material of the 3D-printed models enabled them to have malleable properties that closely resembled kidney tissue, making them useful in surgical simulation for complex cases.
Kusaka et al. 2015 [23]	1 case of a donor graft kidney model and 1 case of a donor kidney and recipient pelvic cavity.	The feasibility and vale of 3D-printed kidney graft models and pelvic cavity replicas for pre-operative and intra-operative simulation in kidney transplantation.	OsiriX (Pixemeo, Geneva, Switzerland) image processing software. Magics (Materialise, Leuven, Belgium) softwar program. Time duration N/A.	CT	Parenchyma printed with VeroClear and TangoPlus material, with VeroMagenta/TangoPlus for the renal artery, VeroCyan/TangoPlus for the renal vein, and VeroMagenta/VeroCyan/TangoPlus blend for urinary tract anatomical structures. TangoPlus for skin layer, and TangoPlus/VeroWhite Plus for pelvic bones, bladder, muscles, and vessels of the pelvic cavity replica. Inkjet printing technology using Connex 500 (Stratasys Ltd., MN, USA) 3D printer. Time duration and cost N/A.	Understanding of spatial relationships amongst vital structures and anatomy was improved using the 3D-printed life-size kidney models due to their accurate replication of anatomy. 3D printing is able to overcome the limitations of viewing 3D anatomy on a 2D monitor and can provide superior depth visualisation. Implementation of 3D-printed kidney models into planning for transplantation surgery may be able to reduce intra-operative complications.
Lee et al. 2018 [24]	10 patients with kidney tumours on the list for robot-assisted partial nephrectomy surgery. 1 surgeon, 1 urologist, 1 resident, and 20 medical students to evaluate models.	Investigate if stereoscopic, 3D-printed customised kidney models can provide superior representations of anatomical structures and be implemented for training of medical students for partial nephrectomy surgery.	Compact View III v.1.03. (Optimum Solution, Korea) software; Blender v.2.76 (Blender Foundation, Amsterdam, NL). Time duration N/A.	CT	Photopolymer material (transparent for renal parenchyma and red for tumour). Objet 260 Connex 3 3D printer (Stratasys, Eden Prairie, MN, USA). USD 650 per model.	Urologist and surgeon group rated the clinical value of the 3D-printed models highly, suggesting they enhance anatomical understanding, facilitate pre-surgical planning, and intraoperative tumour identification (9.9/10, 8.2/10 and 8.4/10, respectively). With the assistance of the 3D-printed models, the student group answered 70% of questions on anatomy, tumour location and morphology correct, compared to 47.3% with only the CT imaging and without the models (*p* < 0.001).
Alyaev et al. 2017 [25]	5 patients with localised RCC.	Investigate usefulness of soft 3D-printed kidney models for treatment and pre-operative planning for patients with localised kidney cancer.	Software and time duration N/A.	CT	Technology N/A.	There was an improvement in intra-operative efficiency utilising the 3D-printed kidney models in pre-operative planning (mean operative time was reduced). Accurate nature of the soft-material, patient-specific 3D-printed kidney models suggests the implementation of 3D-printed organs for surgical training and planning may be of promising use in the future.
Monda et al. 2018 [26]	1 case of a kidney with a tumour 4 cm in diameter. 24 participants (4 medical students, 14 residents, 3 attending surgeons, and 3 endourology fellows) to perform 2 mock surgeries on 2 different days.	Investigate the usefulness of 3D-printed silicone kidney models as an educational resource for inexperienced surgeons.	Invesalius open-source segmentation software (Centro de Tecnologia de Informacao, Amarais, Brazil); Blender (Blender Foundation, Amsterdam, The Netherlands) open-source, 3D-modelling software. Time duration N/A.	MRI	Dragonskin 20 silicone and Slacker silicone deadener (Smooth-On, Inc., Macungie, PA) material. Objet Eden260VS 3D printer (Stratasys, Eden Prairie, MN). 15 min taken per kidney model. 125 g of silicone material used at a cost of USD 3.90 per model.	3D-printed models were useful in improving new technical skills of trainees (mean of 93.8/100) and existing technical skills of trainees (mean of 85.7/100). Silicone material was useful in giving the models tear-resistibility for pre-operative mock surgery to be carried out (needle-driving accuracy mean of 78.3/100 and cutting accuracy mean of 78/100).
Chandak et al. 2019 [27]	3 children < 20 kg referred for renal transplantation.	Assess feasibility of implementing 3D-printed, patient-specific kidney models into pre-surgical planning and practitioner-patient communication for complex cases of pediatric renal transplantation surgery.	Mimics Medical v18.0 software (Materialise NV, Leuven, Belgium); CAD software 3-Matic Medical v.10.0 (Materialise NV, Leuven, Belgium). 4–6 h duration.	CT and MRI	Acrylic polymer material (Objet-Stratasys, Rehovot, Israel). PolyJet technology using Objet500 Connex 1 (Objet-Stratasys, Rehovot, Israel) 3D printer. Cost and time duration N/A.	The kidney models were useful in simulating complex cases such as cases where there was a large size difference between the donor kidney and recipient abdomen, or vascular abnormalities. 3D-printed models improved patient communication, enabling donors to visualise their own and their recipient’s anatomy. 3D-printed models can be incorporated into pre-surgical planning by enabling surgeons to perform a mock transplantation prior to the patient’s transplantation surgery, thereby reducing intra-operative risks.
Von Rundstedt et al. 2016 [28]	10 patients with complex renal tumours.	Investigate if 3D-printed, personalized kidney models with soft-tissue-like properties are able to be used as simulations for robot-assisted partial nephrectomy surgery.	3D Slicer (https://www.slicer.org) editing software; Lazarus 3D (Houston, TX, USA) software. Time duration N/A.	CT and MRI	Mixes of silicone rubber and silicone oil as a thinner (70% by volume rubber and 30% by volume thinner). 3D printer model, time duration and cost N/A.	No discrepancy in surgical resection times between 3D phantoms and real kidneys (6:58 vs. 8:22 min, respectively, *p* = 0.162). 3D-printed kidney models that mimic soft-tissue properties are valuable in their depiction of tumour location, depth, and morphology, suggesting that they may be implemented for pre-surgical rehearsal.
Woliner-van der Weg et al. 2016 [29]	1 case of a kidney and pancreas 3D printed from an 87-kg male patient.	Investigate accuracy and feasibility of 3D-printed phantoms of pancreas and kidneys for use in optimisation of SPECT/CT reconstruction protocols in beta cell imaging using ^111^In-exendin.	Mimics v.14.0 (Materialise HQ, Leuven, Belgium); Meshlab v.1.3.2 (open-source software); SolidWorks v.2012 (Dassault Systemes SolidWorks Corp, Waltham, MA, USA). Time duration N/A.	MRI (T2)	Transparent plastic VeroClear RGD810 material. PolyJet technology using Object Eden250 (Stratasys, Eden Prairie, MN, USA) 3D printer. Time duration and cost N/A.	3D phantom images had similarity to clinical images and showed similar artefacts, with corrections required for pancreas visualisation. The 3D phantoms enabled quantification of pancreatic ^111^In-exendin uptake and selection of the most suitable protocol, alongside the potential for education of clinical ^111^In-exendin image interpretation.
Liu et al. 2018 [30]	2 patients with renal tumours.	Compare the accuracy and associated cost difference of 3D-printed, diseased kidney models printed from a homemade printer vs. a commercial 3D printer.	Analyze12.0 (AnalyzeDirect, Inc., Lexana, KS, USA). 1.5 h time duration.	CT	FLX930 TangoPlus commercial material vs. filament polylactide material for homemade printed models. PolyJet technology using Objet 260 (Stratasys, EDEN 260VS) commercial printer and a homemade 3D printer. 3.5 h taken commercially compared to 4–6 h using homemade printer (per kidney). USD 200 commercially compared to USD 1 homemade.	Good correlation between 2D and 3D images, and 3D-printed models in terms of tumour diameter measurements for both commercially and homemade 3D models (differences less than 0.1 mm). The homemade printer is just as accurate as the commercial printer in its replication of renal anatomical spatial relationships and tumour dimensions, at a much lower cost.
Komai et al. 2016 [31]	10 patients with renal tumours.	Assess 4D navigation experience in minimally invasive off-clap partial nephrectomy procedures utilising 3D-printed kidney models.	CAD software ZedView (LEXI Co. Ltd, Tokyo, Japan) and Freeform (Geomagic, Rock Hill, SC). Time duration N/A.	CT	Acrylic resin material. Biotexture modelling technology using Objet Connex500 (Stratasys, Eden Prairie, MN, USA) 3D printer. USD 450–680 per kidney model. 4–9 h printing duration and 3–9 day duration for total completion of model.	3D-printed models assisted surgeons in visualising the overall kidney anatomical makeup and tumour size and depth, thus facilitating the minimally invasive partial nephrectomy procedure. 3D printed models enhanced patient education on their condition and the planned surgical approach.

Renal Cell Carcinoma (RCC), Three dimensional (3D), Two dimensional (2D), Four-dimensional (4D), Magnetic Resonance Imaging (MRI), Computed Tomography (CT), Single Photon Emission Computed Tomography (SPECT), Not applicable (N/A).
